# Characterization of a beta-glucosidase from *Bacillus licheniformis* and its effect on bioflocculant degradation

**DOI:** 10.1186/s13568-017-0501-3

**Published:** 2017-11-06

**Authors:** Zhen Chen, Tong Meng, Zhipeng Li, Peize Liu, Yuanpeng Wang, Ning He, Dafeng Liang

**Affiliations:** 1 Department of Chemical and Biochemical Engineering, College of Chemistry and Chemical Engineering, Xiamen University, Xiamen, 361005 China; 2 The Key Lab for Synthetic Biotechnology of Xiamen City, Xiamen University, Xiamen, 361005 People’s Republic of China; 3 College of Life Sciences, Xinyang Normal University, Xinyang, 464000 Henan China; 4 Guangxi State Farms Sugar Industrial Group Company Limited, Guangxi Sugarcane Industry R&D Center, Nanning, 530002 Guangxi People’s Republic of China; 5 Guangdong Key Lab of Sugarcane Improvement and Biorefinery, Guangzhou Sugarcane Industry Research Institute, Guangzhou, 510316 Guangdong People’s Republic of China

**Keywords:** β-Glucosidase, Heterologous expression, Polysaccharide bioflocculant, *Bacillus licheniformis*

## Abstract

*Bacillus licheniformis* CGMCC 2876, an aerobic spore-forming bacterium, produces a polysaccharide bioflocculant that is biodegradable and harmless. The present study determined that β-glucosidase played a negative role in bioflocculant synthesis. The gene encoding β-glucosidase was cloned and expressed in *Escherichia coli* BL21. This gene consists of 1437 bp and encodes 478 amino acid residues. The recombinant β-glucosidase (Bgl.bli1) was purified and showed a molecular mass of 53.4 kDa by SDS-PAGE. The expression and reaction conditions of Bgl.bli1 were optimized; the activity of β-glucosidase reached a maximum at 45.44 U/mL. Glucose clearly inhibited the activity of β-glucosidase. The purified recombinant Bgl.bli1 hydrolysed polysaccharide bioflocculant in vitro and synergised with other cellulases. The ability of Bgl.bli1 to hydrolyse polysaccharide bioflocculant was the reason for the decrease in flocculating activity and indicated the utility of this enzyme for diverse industrial processes.

## Introduction

Beta-glucosidase (β-d-glucoside glucohydrolase, EC 3.2.1.21) catalyses the hydrolysis of β-glucosidic linkages of various glycosides and oligosaccharides to form a debranched or shorter oligosaccharide and glucose. In combination with endoglucanases (EC 3.2.1.4) and cellobiohydrolases (EC 3.2.1.91), β-glucosidase can hydrolyse cellulose to glucose (Béguin and Aubert 1994). β-Glucosidase more efficiently stimulates and regulates cellulose hydrolysis by relieving cellobiose inhibition; it is thus a key rate-limiting enzyme of the cellulose-hydrolysing system (Bhatia et al. 2002). β-Glucosidase is distributed throughout a broad range of organisms, including microbes, plants, and animals (Chang et al. 2008; Knight and Dick 2004). Some studies have been conducted to isolate novel β-glucosidases from the genus *Bacillus*, which represents one of the most important groups of bacteria not only for its production of commercially valuable enzymes but also in the study of the mechanism of extracellular polymer secretion (Chamoli et al. 2016; Khan and Husaini 2006; Zahoor et al. 2011).

*Bacillus licheniformis* CGMCC 2876, a Gram-positive and spore-forming bacterium, produces a flocculating agent that is widely used in industrial processes, including wastewater refinement, downstream treatments, and food-related and fermentation processes (Xiong et al. 2010; Zhuang et al. 2012). The bioflocculant from *B. licheniformis* CGMCC 2876 is an extracellular polymer mainly composed of polysaccharide (89%, wt/wt). In the latter phase of polysaccharide fermentation, the flocculating activity decreases because of the degradation of this bioflocculant. At the same time, a gene encoding β-glucosidase (Bgl.bli1) in a bioflocculant-producing clone was predicted to be involved in polysaccharide bioflocculant dissimilation (Yan et al. 2013). The present study describes the cloning, heterologous expression and biochemical characterization of this β-glucosidase. Moreover, the relation between β-glucosidase and polysaccharide bioflocculant was further explored. This report is the first of the hydrolysis of a polysaccharide bioflocculant by β-glucosidase.

## Materials and methods

### Strains, media, and culture conditions

*Bacillus licheniformis* CGMCC 2876 was previously isolated by our laboratory. EPS medium contained (g/L) sucrose or glucose, 13.6; urea, 2.36; MgSO_4_, 0.05; KH_2_PO_4_, 0.04; K_2_HPO_4_, 0.4; and yeast extract, 0.5. The initial pH of the EPS medium was adjusted to 7.2. To produce bioflocculant, conical flasks were incubated at 37 °C on a rotary shaker at 200 rpm for 72 h. *Escherichia coli* BL21 (DE3) cultured on LB medium was used to express Bgl.bli1. Media for *E. coli* BL21 (DE3) transformed with pEASY-E1 was supplemented with 100 μg/mL ampicillin.

### PCR amplification and cloning of *bgl*

The genomic DNA from *B. licheniformis* CGMCC 2876 was extracted using a genomic DNA extraction kit (Omega, China). The gene (*bgl*) encoding β-glucosidase was amplified from the template genomic DNA via a polymerase chain reaction (PCR) using DNA polymerase (Trans, China). The sequences of the oligonucleotide primers used for this gene cloning were based on the DNA sequence of β-glucosidase (GenBank Accession Number: JQ773458). Forward (5′-ATGGCGAGACAAACGTGG-3′) and reverse (5′-TTATTTATACCGGAATTCCTCTGT-3′) primers were designed and synthesized by Sangon (China). The amplified DNA fragment was purified and cloned into a pEASY-E1 6 × His fusion vector using a pEASY-E1 Expression Kit (Trans, China).

### Expression and purification of Bgl.bli1

The resulting recombinant pEASY-*bgl* was transformed into *E. coli* BL21 (DE3). *E. coli* BL21 (DE3) harbouring the recombinant plasmid was grown in an LB-ampicillin medium at 37 °C until the OD_600_ of the culture reached 0.4–0.6, at which point the protein expression was induced through the addition of 0.25–2 mM isopropyl β-d-1-thiogalactopyranoside (IPTG). To obtain maximum expression of the recombinant β-glucosidase, different induction times and concentrations of IPTG were tested. Bacterial cells were incubated for a further 6 h at 37 °C and then harvested via centrifugation at 6000 rpm for 10 min at 4 °C. The cells were washed twice with a solution consisting of 1% Triton X-100 (pH 7.0), 50 mM sodium phosphate and 5 mM EDTA. They were then resuspended in 50 mM sodium phosphate (pH 7.0). The cells were disrupted via ultrasonication (Thermo, MA, USA). The debris and intact cells were removed via centrifugation at 9000 rpm for 10 min at 4 °C to obtain the crude cell extract. The His tag was purified using ProteinIso™ Ni–NTA Resin (Trans, China). The homogeneity of the protein was assessed using 10% SDS-PAGE.

### Enzyme activity and protein concentration assays

Bgl.bli1 activity was determined by measuring the hydrolysis of p-nitrophenyl-β-d-glucopyranoside (pNPG) according to the method in a previous study (Shi et al. 2017). The activity of crude enzyme in the intracellular extract was defined in units per mL of culture. Protein concentrations were determined with a Bio-Rad protein Assay Kit (Bio-Rad, USA) by measuring A_595_ and comparing the results to those of bovine serum albumin standards.

### Determination of flocculating activity

Flocculating activity was measured using kaolin-clay suspensions as an indicator as described previously (Xiong et al. 2010).

### Quantification of reducing sugars

Reducing sugar concentrations were determined by the 3,5-dinitrosalicylic acid (DNS) method. Briefly, 1 mL of appropriately diluted sample solution and 3 mL of DNS reagent were mixed, and the mixture was heated to 99 °C for 5 min in a boiling water bath. Finally, the reducing sugar concentration was calculated from the OD measured at 520 nm and the standard curve. All assays were performed in triplicate, and average values were reported.

### Enzyme characterization

The purified recombinant Bgl.bli1 was characterized as a function of temperature, pH, metal ions and glucose. The optimal temperature for enzyme activity was determined over a temperature range from 30 to 90 °C at pH 7.0 using standard assay conditions. To determine the optimum pH for the recombinant enzyme, we measured β-glucosidase activity over a pH range from 4.0 to 11.0 in increments of 1 pH unit at 50 °C using standard assay conditions. The effects of metal ions on enzyme activity were studied by adding 10 mM CuSO_4_, MgSO_4_, ZnSO_4_, CaCl_2_, NaCl, KCl and MnSO_4_ to separate assay trials. The effect of glucose on enzyme activity was studied by adding 10, 20, 30 and 40% (w/w) glucose. The effects of temperature, pH, metal ions and glucose on enzyme stability were determined by incubating the enzyme under different conditions for periods as long as 0.5 h and then assaying the residual activity.

## Results

### Effect of β-glucosidase on the flocculating activity

**Fig. 1:**
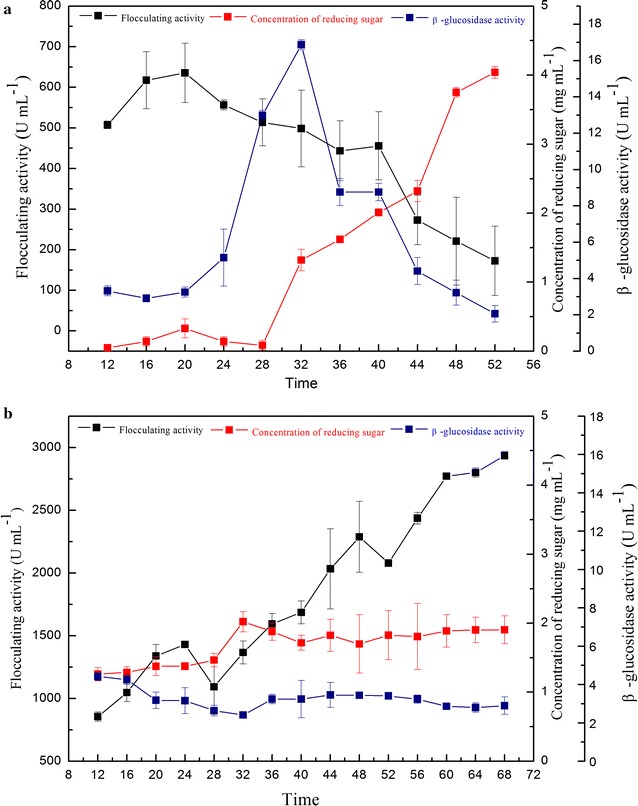
Flocculating activity, β-glucosidase activity and the concentration of reducing sugars **a** using sucrose as substrate and **b** using glucose as substrate

*Bacillus licheniformis* CGMCC 2876 was cultured in a fermentation medium for the production of polysaccharide bioflocculant using sucrose and glucose as carbon sources, and the flocculating activity of the fermentation broth, enzyme activity of β-glucosidase and concentration of reducing sugars were determined during the fermentation period (Fig. 1). When sucrose was used as the substrate, the flocculating activity decreased sharply after 20 h and decreased by as much as half. In contrast, the total enzyme activity of β-glucosidase increased dramatically and reached 16.9 U/mL. The reducing sugar concentration also increased. However, when glucose was used as the substrate, the flocculating activity increased gradually in the late stage, and the changes in the total enzyme activity of β-glucosidase and the reducing sugar concentration in fermentation broth were not significant. The β-glucosidase activity was maintained at a lower level (2–4 U/mL). These results suggested that β-glucosidase activity had a negative effect on flocculating activity. The β-glucosidase was then expressed and purified to further study its functional characteristics.

### Overexpression and purification of recombinant Bgl.bli1

**Fig. 2:**
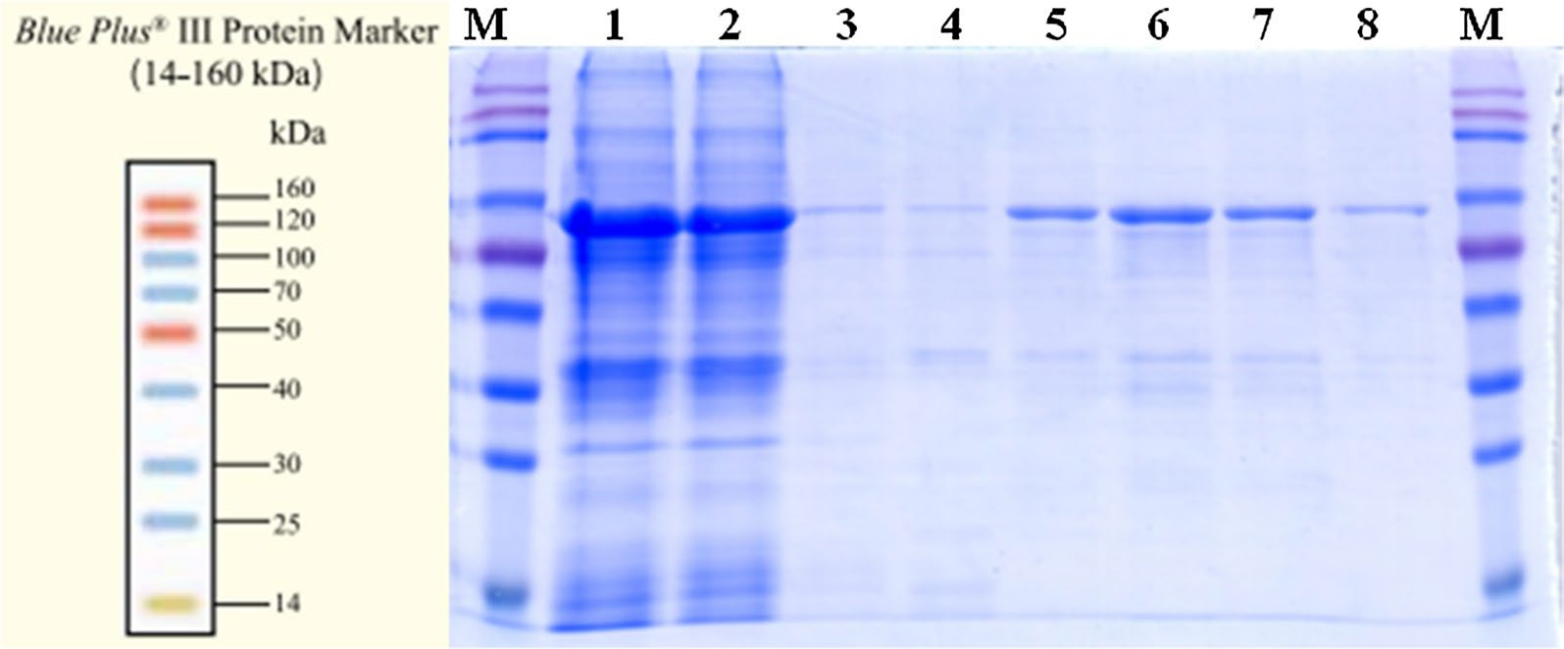
10% SDS-PAGE of Bgl.bli1. Lanes 1 and 2, total protein of cells induced with IPTG; lanes 3–8, purified recombinant Bgl.bli1

The β-glucosidase gene (*bgl*), consisting of 1437 bp, was amplified from the genome of *B. licheniformis* CGMCC 2876 by PCR using the sequence-specific primers described above. Purified PCR product was ligated to *pEASY*-E1, and the resulting plasmid was transformed into *E. coli* BL21. Positive clones were identified by the blue and white screening method. The presence of the *bgl* gene in the recombinant strain was further confirmed by PCR and complete nucleotide sequencing of the insert. The results confirmed that the cloned fragment encoded Bgl.bli1 from *B. licheniformis*. To maximize the yield of the fusion protein in a soluble form, different induction conditions were investigated. Induction with 0.5 mM IPTG at 37 °C and cultivation for 5 h after induction produced the maximum level of soluble, active fusion enzyme, reaching as high as 45.44 U/mL. The recombinant enzyme was purified by Ni–NTA resin, and the supernatant from cell lysates as well as purified protein were then applied to SDS-PAGE (Fig. 2). The molecular mass of the His-Bgl.bli1, calculated via its amino acid sequence, was 53.4 kDa and was identical to the mass obtained by SDS-PAGE. In addition, recombinant Bgl.bli1 represents 46.5% of the total soluble protein in the *E. coli* lysate. This high expression level of the protein in soluble form improves its applicability for industry.

### Characterization of recombinant Bgl.bli1

**Fig. 3:**
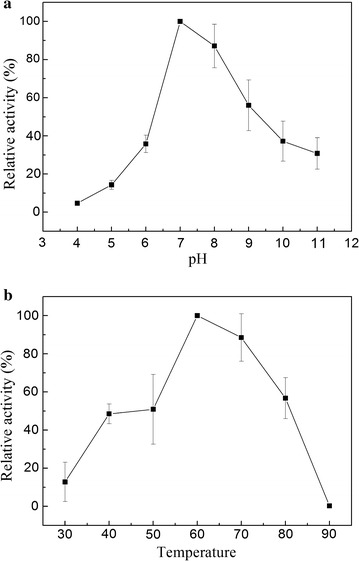
Effects of **a** pH and **b** temperature on the activity of purified recombinant Bgl.bli1

The activity of the purified recombinant Bgl.bli1 was examined over a pH range from 4.0 to 11.0 at 50 °C using phosphate-citric acid buffer. The activity of the recombinant Bgl.bli1 was optimal at pH 7.0 (Fig. 3a). The enzyme activity remained at low levels in acidic solutions, 14.3 and 4.7% of maximal activity at pH 5 and 4, respectively. However, enzyme activity decreased greatly at pH 8.0 and decreased to 56.0% of its maximal level at pH 9.0. These results suggested that Bgl.bli1 is fit for application in neutral or alkaline solutions. Bgl.bli1 showed poor activity at 30 °C. The enzyme activity increased with increasing temperature and reached a maximum at 60 °C. When the temperature was 80 °C, the enzyme retained approximately 80% of its maximum activity, which suggested that Bgl.bli1 is a thermotolerant enzyme with a wide application scope.

The effect of metal ions on recombinant Bgl.bli1 activity was also investigated (Table 1). Bgl.bli1 activity was dramatically increased by the presence of Zn^2+^, Mg^2+^ and Mn^2+^ ions, while Cu^2+^ and Ca^2+^ significantly inactivated the β-glucosidase. Bgl.bli1 activity was not affected by 10 mM Na^+^ and K^+^ ions. These results suggested that the active catalytic domain of the Bgl.bli1 protein contains a divalent metal ion binding site. These properties differed from those of other β-glucosidases from various sources. Jiang et al. (2011) reported that a β-glucosidase, Bgl1E, from uncultured soil microorganisms did not contain a metal ion binding site. (Mallek-Fakhfakh and Belghith 2016) found that a β-glucosidase, Bgl.tls, from *Talaromyces thermophilus* was not affected by the Ca^2+^, Co^2+^, Mg^2+^ and Mn^2+^ divalent metal ions.

**Table 1:** Effects of metal ions on the activity of purified recombinant Bgl.bli1

Solution (10 mM)	Relative activity
NaCl	100.5 ± 3.12
KCl	94.2 ± 2.12
CaCl_2_	80.7 ± 10.62
MnSO4	140.9 ± 0.9
ZnSO_4_	125.3 ± 14.87
CuSO_4_	69.4 ± 1.42
MgSO_4_	127.4 ± 1.52

### The effect of glucose on enzyme activity

Generally speaking, glucose can inhibit the activity of β-glucosidase (Singhania et al. 2013). The inhibitory effect of glucose on the activity of purified recombinant Bgl.bli1 was assayed with various concentrations of glucose. Table 2 shows that Bgl.bli1 activity was gradually repressed with increasing glucose concentration, which revealed that glucose competitively inhibited the β-glucosidase hydrolysis of pNPG. The results confirmed that Bgl.bli1 is a glucose-sensitive glycosidase, which was beneficial to bioflocculant production in *B. licheniformis* CGMCC 2876 because the yield and flocculating activity gradually increased when glucose was used as the carbon source (Fig. 1b).

**Table 2:** Effect of glucose on the activity of purified recombinant Bgl.bli1

Glucose concentration (w/w) (%)	0	10	20	30	40
Enzyme activity (U/mL)	53.31	26.26	20.87	8.67	0.62
Relative activity (%)	100	49.26	39.10	16.26	0

### Hydrolysis of polysaccharide bioflocculant by Bgl.bli1 in vitro

**Fig. 4:**
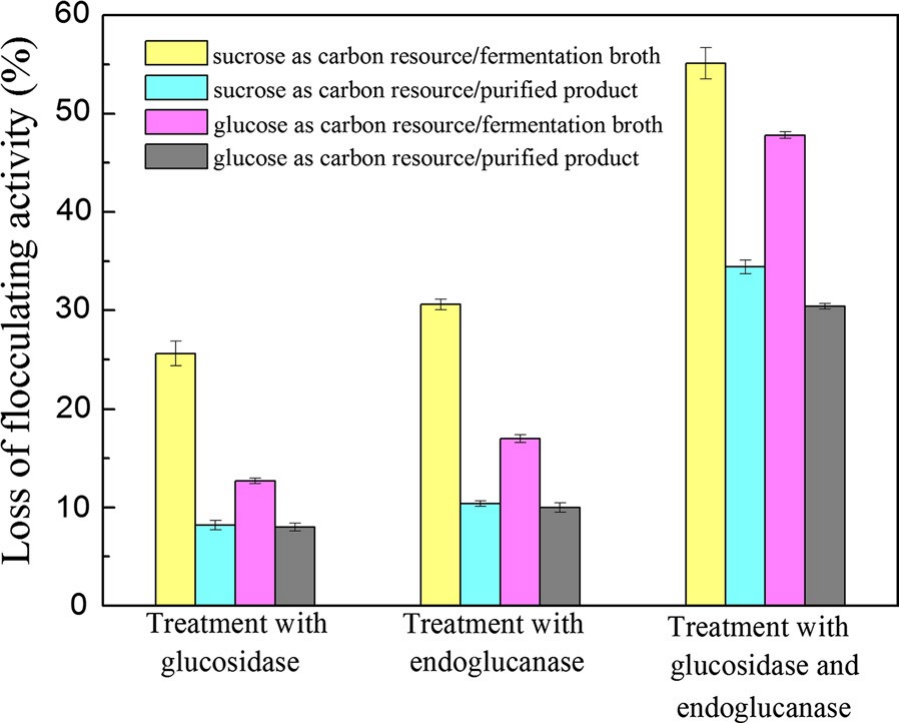
The hydrolysis of polysaccharide bioflocculant by β-glucosidase and endoglucanase

To further verify the hydrolysis of polysaccharide bioflocculant by β-glucosidase, the exopolysaccharide fermentation broth used sucrose or glucose as substrates, and its purified products were treated with crude recombinant Bgl.bli1. The loss of flocculating activity is shown in Fig. 4. When sucrose and glucose are each used as substrates, the flocculating activities of polysaccharide fermentation broth declined by 25.6 and 12.7%, respectively. However, the flocculating activities of purified polysaccharide bioflocculant only decreased by approximately 8%. The different results among samples might be caused by the other glycoside hydrolase in the fermentation broth, which played synergetic roles with β-glucosidase in the hydrolysis of polysaccharide bioflocculant. A commercialized endoglucanase was also used to dispose of the polysaccharide bioflocculant, giving results very similar to those of Bgl.bli1. Furthermore, an enzyme mixture of Bgl.bli1 and endoglucanase was utilized to hydrolyse the polysaccharide bioflocculant, resulting in much greater loss of flocculating activity than when treated with individual enzymes. Bgl.bli1 showed a strong synergistic effect on endoglucanase, with the loss of flocculating activity of purified polysaccharide bioflocculant reaching as high as 30%. The above results suggested that the hydrolysis of the polysaccharide bioflocculant produced by *B. licheniformis* could be improved by the addition of heterologously expressed Bgl.bli1. In other words, Bgl.bli1 played a negative role in the flocculating activity.

## Discussion

Polysaccharide bioflocculant generally has the advantages of high flocculating activity and good thermal stability. However, the flocculating activity of polysaccharide bioflocculant rapidly declines in late fermentation stages. Many polysaccharide bioflocculant-producing strains, including *Virgibacillus* sp. (Cosa et al. 2011), *Bacillus firmus* (Salehizadeh and Shojaosadati 2002) and *Enterobacter aerogenes* (Lu et al. 2005), behave in this manner. Intracellular or extracellular glucoside hydrolase partially or completely hydrolyses the polysaccharide chain (Meng et al. 2015), which could affect the active components of flocculant and result in a decline of flocculating activity. β-Glucosidase was probably a related enzyme that caused the loss of the flocculating activity because of the hydrolysis of the non-reduced end of the cello-oligosaccharide involved in flocculation or because of the relief of substrate inhibition of other cellulases. However, the activity of β-glucosidase was maintained at a low level when sucrose was replaced as the carbon source by glucose. At the same time, the flocculating activity increased continuously in the late stages, instead of decreasing. The result showed that β-glucosidase might be inhibited by glucose in the medium without significantly affecting polysaccharide flocculant activity. This phenomenon was also reported in the transformation of cellulose into glucose (Tokuda et al. 2009). As the glucose concentration in product increased, the activity of β-glucosidase was inhibited, resulting in reduced cellulose degradation. We therefore inferred that β-glucosidase was involved in the degradation of polysaccharide bioflocculant.

In this study, we successfully cloned the *bgl* gene from bioflocculant-producing *B. licheniformis* and achieved a high level of extracellular expression of its protein product in *E. coli*. This strategy can be used for the economical production of β-glucosidase. Further, the recombinant Bgl.bli1 was purified and biochemically characterized in detail for further industrial applications. More importantly, the relation between β-glucosidase and polysaccharide bioflocculant was explored. The β-glucosidase was deemed to decrease the flocculating activity of bioflocculant produced by *B. licheniformis* CGMCC 2876 because of the degradation of polysaccharide bioflocculant. The recombinant Bgl-bli1 showed a strong synergistic effect with an endoglucanase in the hydrolysis of polysaccharide bioflocculant. This study demonstrated that Bgl.bli1 had a negative effect on polysaccharide bioflocculant production when sucrose was used as the carbon source, which would not be a problem when glucose is used as the carbon source, because of glucose sensitivity. Polysaccharide bioflocculant production might be enhanced by knocking out the *bgl* gene. This new discovery will provide fermentation strategies for polysaccharide bioflocculant production by *B. licheniformis*.

## Abbreviations

PCR: polymerase chain reaction
IPTG: isopropyl β-d-1-thiogalactopyranoside
PNPG: p-nitrophenyl-β-d-glucopyranoside
DNS: 3,5-dinitrosalicylic acid
